# Physically Targeted Intravenous Polyurethane Nanoparticles for Controlled Release of Atorvastatin Calcium

**DOI:** 10.18869/acadpub.ibj.21.6.369

**Published:** 2017-11

**Authors:** Behnaz Sadat Eftekhari, Akbar Karkhaneh, Ali Alizadeh

**Affiliations:** 1 Biomedical Engineering Department, Amirkabir University of Technology, Tehran, Iran; 2 Nanotechnology Research Center, Sharif University of Technology, Tehran, Iran

**Keywords:** Drug delivery systems, Nanoparticles, Polyurethanes, Cardiovascular diseases

## Abstract

**Background:**

Intravenous drug delivery is an advantageous choice for rapid administration, immediate drug effect, and avoidance of first-pass metabolism in oral drug delivery. In this study, the synthesis, formulation, and characterization of atorvastatin-loaded polyurethane (PU) nanoparticles were investigated for intravenous route of administration.

**Method:**

First, PU was synthesized and characterized. Second, nanoparticles were prepared in four different ratios of drug to polymer through two different techniques, including emulsion-diffusion and single-emulsion. Finally, particle size and polydispersity index, shape and surface morphology, drug entrapment efficiency (EE), drug loading, and *in vitro* release were evaluated by dynamics light scattering, scanning electron microscopy, and UV visible spectroscopy, respectively.

**Results:**

Within two methods, the prepared nanoparticles had a spherical shape and a smooth surface with a diversity of size ranged from 174.04 nm to 277.24 nm in emulsion-diffusion and from 306.5 nm to 393.12 in the single-emulsion method. The highest EE was 84.76%, for (1:4) sample in the emulsion-diffusion method. It has also been shown that *in vitro* release of nanoparticles, using the emulsion-diffusion method, was sustained up to eight days by two mechanisms: drug diffusion and polymer relaxation.

**Conclusion:**

PU nanoparticles, that were prepared by the emulsion-diffusion method, could be used as effective carriers for the controlled drug delivery of poorly water soluble drugs such as atorvastatin calcium.

## INTRODUCTION

The efforts to improve the important features of nanoparticles such as size, surface properties, and shape have caused the emergence of engineered nanoparticles, which could be used to effectively increase the accuracy of drug delivery by overcoming biological barriers[1,2]. Biological barriers like skin, nasal, small intestine, blood brain barrier, and mouth mucosa limit the delivery of drugs to their desired targets. In administration routes, nanoparticles have overcome these barriers and improve drug bioavaibility, protection of therapeutic agents, and the effectiveness of drug delivery[3]. Atorvastatin calcium (AC) [R-(R*, R*)]-2-(4-fluorophenyl)-b,d dihydroxy-5-(1-methylethyl)-3-phenyl-4-[(phenylamino) carbonyl]-1H-pyrrole-1-heptanoic acid calcium salt (2:1) trihydrate is a member of statin family that is widely used to reduce cholesterol levels, thereby preventing cardiovascular diseases, breast cancer metastasis, inflammatory colitis, chronic renal disease, and nasal polyp disease and to treat Alzheimer’s disease[4,5]. Because of hydrophobic nature and high molecular weight of AC, the absorption and bioavailability of this drug are affected by two important factors in the oral route: (1) the presystemic clearance in the gastrointestinal mucosa, (2) extensive first pass metabolism in the liver. Also, this drug is associated with serious adverse effects like rhabdomyolysis on chronic administration[6,7]. Intravenous administration is commonly used to avoid drug metabolism, to increase bioavailability of drug and to control the rate of distribution. According to the aforementioned reasons, there is a need to synthesize an injectable nano-drug delivery system for targeted and controlled release of AC to develop a new efficient therapeutic approach for this drug.

Polyurethanes (PUs) are formed by step polymerization between isocyanates and polyols to yield polymers with urethane bonds (–NH–COO) in their main chain[8]. The great variety of building blocks allows the chemical and physical properties of PUs to be appropriate for specific target applications, particularly for biomaterials and pharmaceutical fields and enhance the effectiveness of the loaded drug[9,10]. Medical PUs have some specific properties such as biocompatibility and mechanical flexibility. Bioiner PUs are often used as catchers, heart valves, vascular graft, prostheses, and other blood contact devices due to their chemical stability, abrasion resistance, and appropriate mechanical properties[11,12]. There are hydrolysable linkages in biodegradable PUs structure (e.g polyester urethane and polyether urethane). Such linkages are suitable for drug delivery systems and scaffolds in tissue engineering[13,14].

In this study, polycaprolactone (PCL) diol (MW: 2000 Da), hexamethylene diisocyanate (HMDI) and 1,4-butanediol (BD) were used for PU synthesis. This kind of PU has several attributes such as biocompatibility, hemocompatibility, biodegradability, and excellent mechanical properties. Due to these advantages, PU-based nanoparticles are widely utilized for controlled delivery of proteins, growth factors, antibiotics, antitumor drugs, and other bioactive substances[15].

Generally, six main techniques are employed for nanoparticles preparation: nanoprecipitation, emulsion-diffusion, single-emulsification, emulsion-coacervation, polymer-coating, and layer-by-layer methods[16]. These techniques provide major alternations in structure, composition, and physico-chemical properties of nanoparticles. The main reasons for choosing emulsion-diffusion and single-emulsion methods for AC encapsulation are: suitability for encapsulation of hydrophobic drug, stability of drugs, feasibility of method, low time consumption, and low generation of contamination[17,18].

This study was based on at least two facts. First, the higher bioavailability of AC in injection administration in comparison to the oral form. Second, the excellent properties of PU, such as blood compatibility, as a carrier for this administration. Therefore, in this study, injectable PU-based nanoparticles were prepared by both emulsion-diffusion and single-emulsion methods in order to control the release of AC. Then the effect of preparation parameters on the characteristics of nanoparticles, including size, charge, drug loading (DL), encapsulation efficiency, and *in vitro* drug release was investigated. To the best of our knowledge, there has been, so far, no report on utilizing this system in the literature.

## MATERIALS AND METHODS

### Materials

AC was a kind gift from the Chemidarou Pharmaceutical Company, Tehran, Iran. PCL diol with an average molar mass of 2000 g/mol, HMDI (168.2 g/mol), BD (90.18 g/mol), acetone, polyvinyl alcohol (PVA, 31000 g/mol), and chloroform were obtained from Sigma Aldrich, Germany. All other chemicals were of analytical reagent grade.

### Polymerization and preparation of polyurethane

**Fig. 1:**
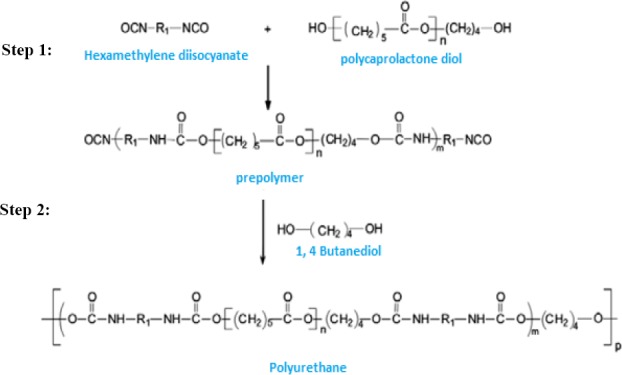
Polymerization step for synthesizing polyurethane. Step 1, the prepolymer form by hexamethylene diisocyanate and polycaprolactone diol reaction; Step 2, prepolymer reaction by 1,4-butanediol for polyurethane synthesis.

PU was synthesized from PCL diol and HMDI via a condensation reaction as shown in Figure 1[19]. PCL, HMDI, and BD were used in stoichiometry ratio. The ratio of urethane groups (NCO/OH) was 1:1. PCL diol was dried in a vacuum oven at 60°C for 5 hours. PCL was then dissolved in 30 ml acetone in a 250-ml glass reactor. For synthesis of prepolymer, HMDI was added to glass reactor drop-wise, and BD was added to the solution drop-wise after two hours. The mixer was stirred at 400 rpm in the oil bath under nitrogen gas throughout the work. After two hours, the polymer solution was heated at 56°C under vacuum for solvent evaporation.

### Nanoparticles synthesis using emulsion-diffusion method

AC-loaded PU nanoparticles were prepared by emulsion-diffusion method as described by Miladi *et al*.[20]. In the first step, four different weight ratios of drug to polymer (1:1, 1:2, 1:3, and 1:4) were dissolved in 10 ml acetone. The oil solution was then dispersed drop-wise in the aqueous phase containing 0.5% PVA, as the stabilizer, by a homogenizer under 15000 (rpm). The obtained suspension was stirred to evaporate solvent for 2 hours. Subsequently, nanoparticles were washed with deionized water and centrifuged at 44800×g for 10 minutes, and the supernatant was

### Nanoparticles synthesis using single-emulsion method

Nanoparticles were prepared by single-emulsion technique according to Rosca *et al*.[21]. In this method, four various ratios of PU to AC were dissolved in 10-ml chloroform. PU amounts were used to provide drug to polymer weight ratio 1:1, 1:2, 1:3, and 1:4. This organic phase was added to the water phase containing 0.5% PVA. This emulsion was homogenized at 15000 rpm for 5 minutes. Solvent evaporation was performed by stirring the emulsion for three hours. Nanoparticles suspension was centrifuged at 44800 ×g, and the supernatant was collected. Freeze-dried nanoparticles were put in a desiccator containing silica gel.

### Fourier Transform Infrared Radiation (FTIR) Measurement

FTIR analysis was carried out for synthesized PU and atorvastatin-loaded PU nanoparticles using KBr pellet method on a FTIR spectrophotometer (Thermonicolet NEXUF 870, USA). Scans for samples were recorded at a resolution of 2 cm^−1^ over the wavenumber region of 4000-400 cm^-1^.

### Proton nuclear magnetic resonance (^1^H-NMR) spectra

Proton nuclear magnetic resonance was obtained with a 300 MHz Varian spectrometer (Palo Alto, CA, USA) by using acetone as the solvent.

### MTT cell proliferation and viability assay

Extraction process was carried out according to the standard ISO 10993-5:2009; to evaluate the toxicity of PU and its influence on the growth and proliferation of HEK293 cells. First, five samples of 0.0000375, 0.0000750, 0.0001125, 0.000150, and 0.0001875 mg were transferred to a sterile 24-well plate. Thease amounts of polymer are suitable for synthesis of polyurethane nanoparticle. Next, 700 λ medium was added to each well, and the samples were incubated for 15 days. MTT reagent (10 µl) was added to each well and was further incubated for 24 hours. The plate was kept in an incubator under controlled conditions at 37°C for 24 hours. After 24 hours, the culture supernatant was removed, and a new medium with yellow MTT solution (3[4,5-dimethylthiazol-2-yl]-2,5-diphenyl tetrazolium bromide; a tetrazole) was added to the samples. The samples were then incubated for four hours. After incubation, MTT solution was removed and the insoluble formazan crystals were dissolved by adding 2-propanol. The absorbance of the colored solution was obtained by a spectrophotometer measuring at λ=570 nm and compared with the negative control sample (without material)[22].

### Measurement of particle size, polydispersity index (PDI), and zeta potential of prepared nanoparticles

Particle size, distribution of particle size (PDI), and zeta potential of PU nanoparticles were measured by photon correlation spectroscopy using zetasizer (Malvern, UK). Samples were diluted appropriately with the aqueous phase of the formulation. Particle size analysis was performed at room temperature.

### Determination of entrapment efficiency (EE) and drug loading

The amount of atorvastatin encapsulation was determined by using the indirect method. For this purpose, prepared nanoparticles were centrifuged at 44800 ×g for 15 min. Then the supernatant was collected, and the amount of drug was measured by an ultraviolet spectrophotometer at 246 nm (Milton Roy Spectronic60, USA). DL and encapsulation efficiency were calculated according to the following equations [20].


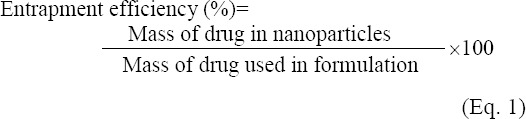



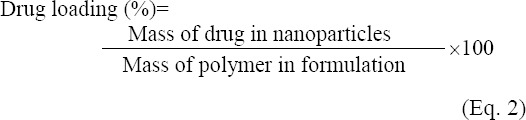


### Scanning electron microscopy (SEM)

Shape and surface morphology of nanoparticles was evaluated by SEM.

### In vitro release studies

Nanoparticles were dispersed in 10-ml phosphate buffer pH 7.4, at 37°C. At the same intervals, all of the released media were recovered and replaced with 10 ml fresh medium to sustain sink condition. After centrifugation, the amount of released drug was measured by UV spectrophotometry.

### Statistical analysis

All the statistical analyses in this study was performed using SPSS 15.0 software (SPSS Inc., US). The level of significance for all statistical analyses was set at *P*<0.05.

## RESULTS AND DISCUSSION

### Fourier transform infrared spectroscopy

**Fig. 2:**
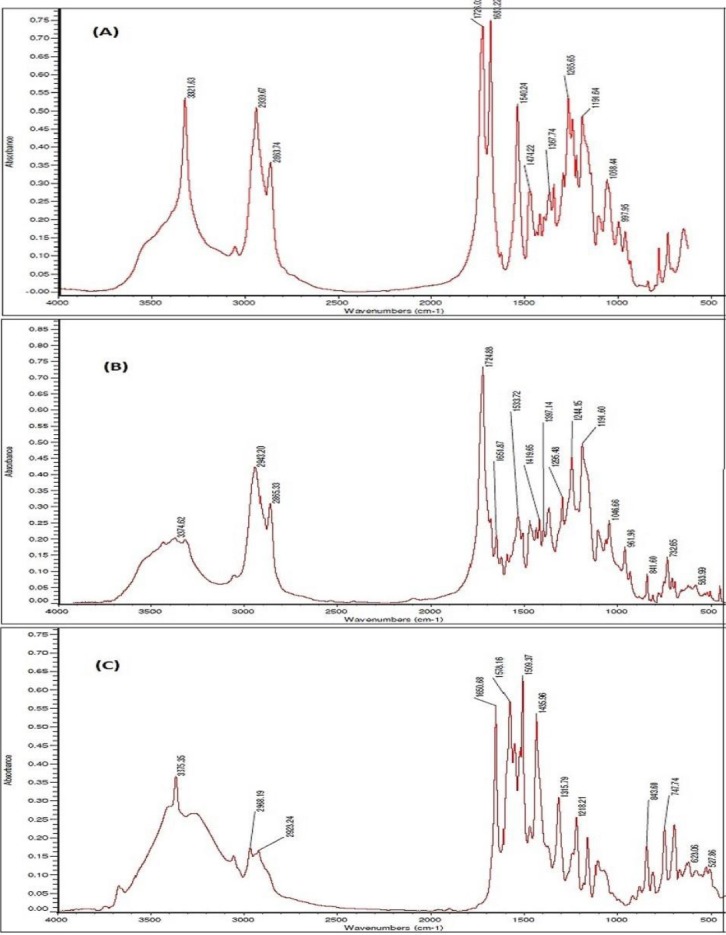
Fourier Transform Infrared Radiation (FTIR) spectra. FTIR spectra of synthesized polyurethane (A), atorvastatin calcium (B), and AC-loaded polyurethane (C).

The formation of PU was investigated by FTIR spectra (Fig. 2A). Since the absorption band was not observed in the 2200-2300 cm^−1^, we ensured that the reaction of NCO groups is complete, and there is no free NCO in the polymer, and the purification steps were completed successfully. On the other hand, if a small amount of moisture is present in the reaction system, the isocyanate groups can react with water molecules to form urea connections. Because of these phenomena, a poor absorption peak will be observed at 1620 cm^−1^in the FTIR spectrum. Due to the lack of this peak in the spectrum of the synthesized sample, it can be asserted that the reaction is completely devoid of moisture, and all isocyanate groups are used for the formation of urethane groups. The existence of absorption peaks at 1726 cm^−1^ and 3321 cm^−1^ are related to carbonyl (C=O) and (N-H) groups, respectively. As a result, this spectrum pattern confirms the PU formation[19].

FTIR spectra of pure AC and atorvastatin-loaded nanoparticles are shown in Figure 2B and 2C. Indicator peaks of atorvastatin structure include stretching absorption band of NH and carbonyl (C=O), stretching absorption band at 3374 cm^−1^ and at 1651 cm^−1^, respectively[7]. These peaks are shown in nanoparticles spectra at 3375 cm^−1^ and 1651 cm^−1^, respectively. Slight displacement of these peaks indicates the lack of interaction between drug and PU.

### Nuclear magnetic resonance

**Fig. 3:**
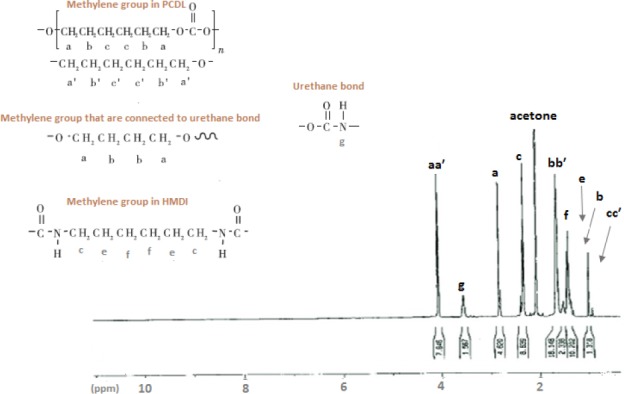
^1^H -NMR spectrum of synthesized polyurethane. We can observe methyl protons internal to the polyurethane chains at 3.7 ppm (g); the methyl protons belonging to the units directly bonded to the urethane groups at 2.9 (a) and 1.2 (b). Methylene groups in polycaprolactone diol are shown at 4.05 (aa′), 1.9 (bb′), and 1.2 (cc′). The methylene groups were directly bonded to hexamethylene diisocyanate present at 2.7 (c), 1.2 (e), and 1.6 (f).

Figure 3 shows the proton (^1^H-NMR) of synthesized PU. Electron factors and different functional groups caused the chemical shift of proton in the polymer bulk. Chemical shift in 4.055-1.677-1.420, 2.835-1.416, 2.340-1.637-1.435, and 3.731 ppm regions are corresponded to CH3 and CH2 in the PCL, the methylene group, that are connected to urethane bond, a methylene group in HMDI and urethane bond in spectra, respectively[15].

### MTT assay

**Fig. 4:**
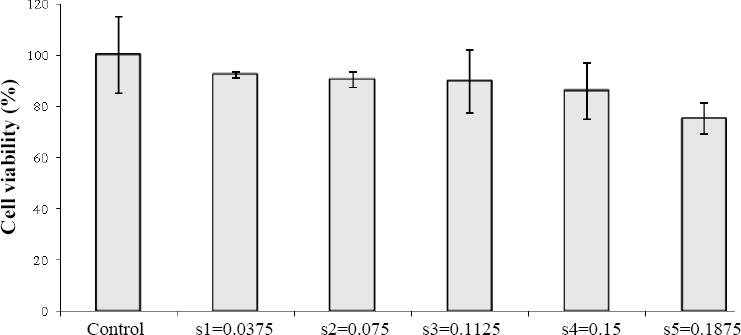
Cell viability measured by MTT assay. The percentage of live cells is reduced as the polymer concentration increases.

The biocompatibility of the synthesized PU was assessed by HEK293 cells from the human embryonic kidney. The resulted graph is shown in Figure 4. However, the percentage of live cells was reduced as the polymer concentration was increased. However, for all samples, cell viability is more than 75%, showing the ideal biocompatibility of synthesized PU after the incubation with the human cells[9].

### Particle size, polydispersity index, and zeta potential

The features of AC-loaded nanoparticles prepared by emulsion-diffusion and single-emulsion are shown in Tables 1 and 2, respectively. The size of nanoparticles varies from 174.04±2.18 nm to 277.24±9.24 nm for emulsion-diffusion and from 306.5±6.13 nm to 393.12±3.34 nm for single-emulsion. The solvent used in emulsion-diffusion was a water-miscible solvent such as acetone, while chloroform was used in single-emulsion, as a water-immiscible solvent. The size of nanoparticles prevents the aggregation of emulsion droplets, which in other words provides emulsion stability[18]. Small particle size in emulsion-diffusion method is ascribed to the protective role of emulsion stabilizer against coagulation of particles, as well as low interfacial tension between the organic solvent and aqueous phase due to acetone water-miscible nature. Chloroform immiscible nature and its organic chemical structure make PVA not to be able to completely prevent the fusion and integration of particles. Thus, synthesized nanoparticles are larger than single-emulsion method nanoparticles.

**Table 1:** Characteristics of nanoparticles prepared by emulsion-diffusion

Sample code	PDI	Size (nm)	Zeta potential (mV)	Entrapment efficiency (%)	Drug loading (%)
DE(1:1)	0.029±0.013	174.04±2.18	-3.97±0.05	68.00±0.23	68.00±0.54
DE(1:2)	0.005±0.002	207.16±5.02	-5.79±0.12	74.93±0.42	37.46±0.77
DE(1:3)	0.001±0.001	220.02±3.12	-8.04±0.09	80.85±0.35	26.93±0.29
DE(1:4)	0.004±0.003	277.24±9.24	-9.48±0.04	84.76±0.39	21.18±0.38

PDI, polydispersity index

**Table 2:** Characteristics of nanoparticles prepared by single-emulsion

Sample code	PDI	Size (nm)	Zeta potential (mV)	Entrapment efficiency (%)	Drug loading (%)
SE(1:1)	0.009±0.002	306.5±6.13	-2.08±0.02	26.53±0.63	18.13±0.39
SE(1:2)	0.007±0.004	327.4±2.23	-3.38±0.09	31.68±0.26	13.22±0.43
SE(1:3)	0.014±0.007	342.01±9.20	-5.48±0.10	37.65±0.42	10.41±0.51
SE(1:4)	0.008±0.005	393.12±3.34	-8.72±0.07	42.11±0.37	10.02±0.18

PDI, polydispersity index

**Fig. 5:**
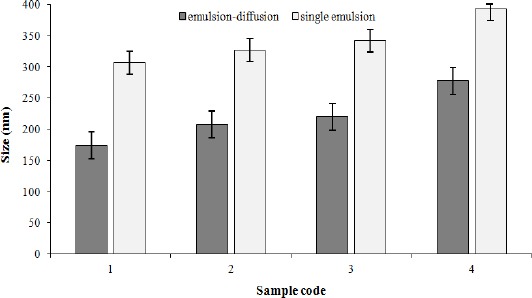
Influence of drug to polymer ratio and solvent type on particle size. In both methods, the increase of the polymer in samples and therefore the decrease of the drug to polymer weight ratio from 1:1 to 1:4 resulted in a significant enhancement of particle size (*P*<0.05).

It is obvious from Figure 5 that in both methods the increase of the polymer in samples and therefore the decrease of the drug to polymer weight ratio from 1:1 to 1:4 resulted in a significant enhancement of particle size (*P*<0.05(. This finding could be explained by the increase of organic phase viscosity and the droplet size of emulsion following PU concentration increase. Particle size and its distribution depend on net shear force that enters the organic phase during nanoparticle formation[23]. Thus, by increasing the viscosity, shear force is reduced and particle size increases. Also, reinforcement in the organic phase and viscosity, due to the presence of hydrophobic-hydrophobic bond, creates a barrier against penetration of polymer from the organic phase to the aqueous phase.

Zeta potential ranged from -2.08±0.02 mV to -9.48±0.04 mV. This action is a function of nanoparticles surface charge, absorbed layer on the surface of the nanoparticles on the border between the suspension and nanoparticles, as well as the nature and structure of the suspension environment[24]. PU exhibited a negative value of zeta potential due to the superficial availability of COOH groups[25]. In emulsion-diffusion method, zeta potential was decreased slightly from -3.97±0.05 mV to -9.48±0.04 mV, while drug to polymer ratio was increased from 1:1 to 1:4. These results indicated that the drug to polymer ratio did not affect zeta potential.

Low PDI of samples showed that the distribution range of particle size was narrow. It has been reported that PDI values of lower than 0.1 are indicative of reducing the aggregation of particles[25]. As it is apparent from Tables 1 and 2, the PDI for all samples are lower than 0.1, and the best results were achieved in emulsion-diffusion method.

### Determination of entrapment efficiency and drug loading

As seen in Tables 1 and 2, in single-emulsion method, EE% and DL% are considerably lower than emulsion-diffusion method. The highest EE% were 84.76±0.39% and 42.11±0.37% both for 1:4 ratio in the emulsion-diffusion and single-emulsion techniques, respectively. Because of the hydrophobic nature of AC and its significant solubility in chloroform organic solvent, this drug tends to be carried alongside chloroform during slow elimination of solvent from the oil phase to the aqueous phase. Nevertheless, it should be thought that the slight polarity of AC structure is another cause for drug permeation into the water phase. Hence, the encapsulation of drug is decreased in single-emulsion method. When acetone is used as miscible-water solvent, emulsification step is removed due to the immediate solvent evaporation. Therefore, the hydrophobic structure of atorvastatin entraps this drug in quickly formed nanoparticles.

**Fig. 6:**
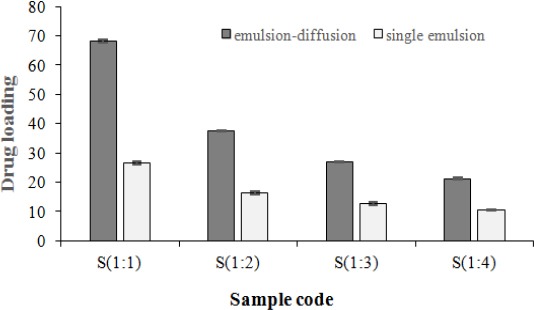
Influence of drug to polymer ratio and solvent type on entrapment efficiency. The encapsulation efficiency in emulsion-diffusion and single-emulsion techniques was increased respectively from 68±0.23% and 26.53±0.63% to 84.76±0.39% and 42.11±0.37%, while drug to polymer ratio was augmented from 1:1 to 1:4) *P*<0.05).

Figure 6 demonstrates that encapsulation efficiency in emulsion-diffusion and single-emulsion techniques was increased respectively from 68±0.23% and 26.53±0.63% to 84.76±0.39% and 42.11±0.37%, while drug to polymer ratio was augmented from 1:1 to 1:4 (*P*<0.05(. Poor water solubility of AC resulted in the enhancement of the binding between polymer and drug, which enables entrapping of higher amount of drug into nanoparticles by increasing the polymer. In addition to drug encapsulation, absorption of drug on the surface of the nanoparticle causes the enhancement of DL%. For all samples, DL% was reduced via a decrease in drug to polymer ratio for both methods. For all samples, DL% was decreased via reduction drug to polymer ratio for both methods because of the ratio of drug mass to total nanoparticle decrease (Tables 1 and 2). High quantity of drug in the core nanoparticles decreases the initial burst release of drug and prolongs the time of drug release[20].

### Morphological characterization of prepared nanoparticles

**Fig. 7:**
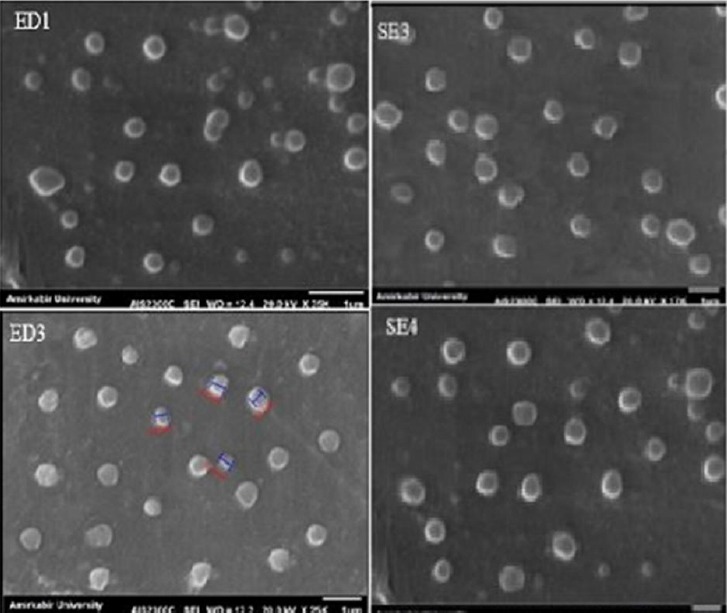
SEM image of selected formulations. The scale bar represents 1 μm.

Surface morphological features of AC-loaded PU nanoparticles were analyzed by SEM (Fig. 7). In both methods, the prepared nanoparticles had a smooth surface and a spherical shape, and SEM showed that the distribution of particle size is narrow.

### Physical properties of nanoparticles and therapeutic effect

**Fig. 8:**
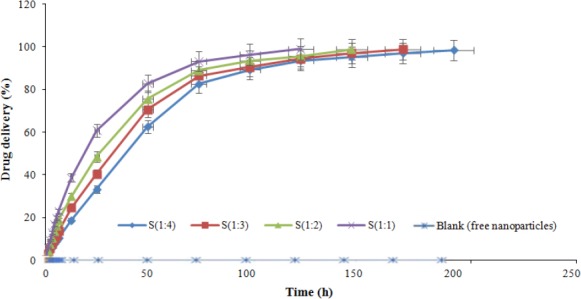
*In vitro* release profiles of atorvastatin calcium from polyurethane-based nanoparticle in phosphate buffer (pH 7.4).

It has been reported that physical properties of nanoparticles are in correlation with their healing effect[1]. The size of nanoparticles may affect their biological properties such as immunogenicity, circulation time, cellular uptake, as well as half-life and degradation. As an example, because of large numbers of vascular fenestrations around tumors, the extravasation of nanoparticles with the size ranging from 100 to 200 nm will occur[2]. Therefore, as it is observed in Table 1, DE1 formulation with 174.04±2.18 nm is more effective for the release of AC in the medium of breast cancer tumors. According to the fact that the particles with the size larger than 200 nm accumulate in the liver, in order to reduce the cholesterol level, DE2, DE3, and DE4 formulations are suitable for controlled delivery of atorvastatin in this organ. Also, surface charge and shape of nanoparticles influence the cellular interactions, macrophage uptake, and opsonisation. For example, spherical negatively charged nanoparticles have longer circulation lifetimes and less uptake in the macrophages[2]. In this study, the generated nanoparticles show a high potential for prolonged circulation and less immunogenicity (Table 1 and Fig. 8).

### In vitro release studies

Having the appropriate size and drug encapsulation of prepared nanoparticles by emulsion-diffusion method, the *in vitro* release of AC was investigated for these samples. Obtained release data are shown in (Fig. 8). Drug release from nanoparticles were evaluated for eight days, and drug release profile reflected the ability of nanoparticles to have a sustained release of AC after 24 h. These profiles show that as polymer amount is increased, drug release becomes more sustained. Kinetic profiles of AC release for all samples could be characterized by first rapid phase (burst release), followed by second slower phase (controlled profile release).

The rate of drug release from specific polymer nanoparticle loaded with the same drug is related to many factors such as the molecular weight of polymer, polymer degradation, polymer concentration, solubility of the drug in polymer, particle size, morphology, and preparation condition (pH and temperature of medium)[23]. The absorbed drug on nanoparticles surface could be increased by decreasing the size of particles. This initial burst phenomenon might be due to the rapid release of AC deposited on the surface and in the water channels of nanoparticles. Owing to the mentioned factors, the amount of burst release from DE1, DE2, DE3, and DE4 was found to be 77.36%, 59.86%, 49.32%, and 37.48%, respectively. Also, the results indicated that drug-polymer ratio governed the drug release from these nanoparticles. Drug release rates were decreased with increasing amounts of AC in the formulation. These controlled drug delivery systems elevate the bioavailability of AC by improving its solubility.

To gain insight into the mechanism involved in synthesized nanoparticles release process, the obtained data were analyzed by standard *in vitro* release models, and the value of resulting regression coefficient was calculated (Table 3). The drug release data compliances with Korsmeyer–Peppas, which is a semi-empirical model that describes the drug release from a polymeric system and is presented by following equation[25]:


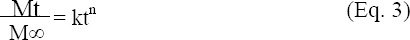


**Table 3:** Curve fitting analysis of AC-loaded particles

Sample code	R^2^	n
DE(1:1)	0.9764	0.7871
DE(1:2)	0.9899	0.7894
DE(1:3)	0.9867	0.8324
DE (1:4)	0.9993	0.8381

In all cases, release kinetics fitted the Korsmeyer-Peppas model with R^2^ values higher than 0.97, which suggests that active release was mainly controlled by diffusion and polymer relaxation.

Where *M*_t_ and *M*_∞_ are the absolute cumulative amount of drug released at time t and at infinite time, k is constant depending on chemical structure and geometry of system, and *n* is the value of release and is indicative the release mechanism. In this model, n value varies between 0.43 and 0.85 for controlled drug delivery with release mechanism of diffusion and polymer relaxation. This statement means that the drug release is controlled by non-Fickian diffusion method. However, if n=0.43, this indicates that drug release is governed by Fickian diffusion method. Diffusional release is associated with molecular diffusion of the drug due to a chemical potential gradient. Also, n≥0.85 implies a relaxation release, which occurs by swelling of polymer in water or biological fluids[26]. As indicated in Table 3, for all cases, the release kinetic data were fitted with Korsmeyer-Peppas model with R^2^ values higher than 0.9743, and n values were in the range of 0.7871-0.8381. Based on these results, mechanisms of AC release from PU nanoparticles include two phenomena, drug diffusion and polymer chain relaxation. These mechanisms are in accordance with the result of drug release profiles.

We synthesized PU-based nanoparticles loaded with AC, which is a hydrophobic drug for treatment of hypercholesterolemia and prevention of breast cancer metastasis diseases. For this purpose, PU was successfully synthesized by using HMDI, PCL diol, and BD. Nanoparticles were prepared by two methods: emulsion-diffusion and single-emulsion. It was found that preparation method and organic solvent had a great effect on characteristics of the prepared nanoparticles. In addition, for all samples, SEM images showed a smooth surface in accordance with PDI results with narrow distribution. Obtained nanoparticles with emulsion-diffusion have improved characteristics such as smaller size, lower size distribution, higher drug encapsulation, and targeted drug release due to their suitable size, zeta potential, and shape. The EE for this method was 68±0.23% to 84.76±0.39%. *In* *vitro* results from nanoparticles prepared by emulsion-diffusion methods nanoparticles are in agreement with Korsmeyer-Peppas model, reflecting diffusion and polymer relaxation. Furthermore, we found that the PU nanoparticles prepared by emulsion diffusion technique are suitable controlled delivery systems for hydrophobic drugs such as AC.
